# The Correlation Between Functional Movement Screen Scores and Self-Reported Injury History Among Competitive Male Padel Players: A Cross-Sectional Study

**DOI:** 10.3390/sports14050208

**Published:** 2026-05-18

**Authors:** Khalid Yaseen, Mohannad Felemban, Layan Barassin, Elan Alnakeeb, Anfal Astek, Ziyad Neamatallah, Mazen Homoud, Khalid Alsayed, Mishari Rowished, Mazen Almutairi, Ayah Ismail

**Affiliations:** 1 Department of Physical Therapy, Faculty of Medical Rehabilitation Sciences, King Abdulaziz University, P.O. Box 80324, Jeddah 21589, Saudi Arabia; mffelemban@kau.edu.sa (M.F.); layan.br.2000@gmail.com (L.B.); elan.alnakeeb@icloud.com (E.A.); aastek@kau.edu.sa (A.A.); zneamatallah@kau.edu.sa (Z.N.); mishari.row@gmail.com (M.R.); mazen.almutairi77@gmail.com (M.A.);; 2 Department of Respiratory Therapy, Faculty of Medical Rehabilitation Sciences, King Abdulaziz University, P.O. Box 80324, Jeddah 21589, Saudi Arabia; mhomoud@kau.edu.sa; 3 Department of Prosthetics and Orthotics, Faculty of Medical Rehabilitation Sciences, King Abdulaziz University, P.O. Box 80324, Jeddah 21589, Saudi Arabia; kaalsayd@kau.edu.sa

**Keywords:** Functional Movement Screen, injury history, padel, movement quality, competitive athletes

## Abstract

**Background:** Padel is a rapidly growing sport, yet limited evidence is available regarding movement quality and injury history among competitive players. The Functional Movement Screen (FMS) may help describe movement patterns associated with previous injury, although its predictive value remains uncertain. This study examined the association between FMS total and component scores and self-reported injury history among competitive male padel players. **Methods:** A cross-sectional study was conducted involving 17 competitive male padel players, with 9 injured and 8 uninjured based on self-reported musculoskeletal injury history within the preceding 12 months. Movement quality was assessed using the seven-item FMS. Spearman’s rank correlation was used to examine the association between FMS total score and injury history, while Mann–Whitney U tests were used to compare FMS total and component scores between groups. The seven component-level comparisons were considered exploratory. Bonferroni correction was applied by using an adjusted significance threshold of α = 0.05/7 = 0.007; therefore, unadjusted *p*-values were interpreted against this corrected threshold. **Results:** Lower FMS total scores were associated with previous injury history (ρ = −0.703, 95% CI: −0.89 to −0.38, *p* = 0.002). Previously injured players demonstrated lower FMS total scores than uninjured players (*p* = 0.005). Among individual components, the In-Line Lunge showed a significant between-group difference after Bonferroni correction (*p* = 0.004), suggesting lower performance in a task requiring lower-limb stability, mobility, and trunk control. **Conclusions:** In this small exploratory cross-sectional study, lower FMS scores were associated with self-reported previous injury among competitive male padel players. These findings should be interpreted cautiously, as the study design does not allow causal or predictive conclusions. Larger prospective studies are needed to clarify whether FMS scores have practical value in monitoring movement quality in padel athletes.

## 1. Introduction

Padel is a rapidly growing sport worldwide, particularly in Europe and the Middle East, characterized by a unique combination of tennis and squash, and predominantly played in a doubles format on enclosed courts [1]. The sport places high physical demands on players due to repetitive high-intensity actions, including short sprints, rapid accelerations and decelerations, abrupt stops, and frequent multidirectional movements, primarily in the forward and lateral planes [1,2]. These biomechanical demands expose players to considerable musculoskeletal stress and increase susceptibility to injuries. The injury profile of padel players reflects the repetitive and asymmetric nature of the sport. Epidemiological studies have identified the elbow as the most commonly injured anatomical site, and knee injuries as the second most prevalent injury site associated with repetitive lateral movements, cutting actions, sudden changes in direction, and impact loading, leading to ligamentous or meniscal pathologies [2,3].

Given the injury burden in padel, effective screening tools that identify athletes at increased risk of injury are essential. The Functional Movement Screen (FMS) is a widely used assessment tool designed to evaluate fundamental movement patterns, identify functional limitations, movement asymmetries, and compensatory strategies [4,5]. The FMS consists of seven standardized movement tasks (Deep Squat, Hurdle Step, Inline Lunge, Shoulder Mobility, Active Straight Leg Raise, Trunk Stability Push-Up, and Rotary Stability), providing a composite score that reflects overall movement quality. A distinctive feature of the FMS is its ability to detect bilateral asymmetries, which are considered clinically relevant risk factors for musculoskeletal injury.

The predictive value of the FMS has been investigated across multiple sports. In football, lower FMS scores have been associated with a higher likelihood of sustaining injuries over a competitive season [6]. Similar findings have been reported in athletes across sports such as basketball, soccer, and volleyball, where athletes scoring below 14 on FMS demonstrated an increased risk of injury [7,8]. In racket sports, including tennis, the FMS has been shown to be effective in identifying movement dysfunctions and guiding corrective exercise programs [9]. Furthermore, incorporating athlete’s previous injury history alongside FMS assessment is particularly important, as prior injury is one of the strongest predictors of future injury occurrence [10].

Despite the growing body of literature on FMS, its application in Padel athletes remains limited. While the movement demands of padel share similarities with other sports where FMS has demonstrated utility, there is a notable lack of evidence examining the relationship between FMS scores and injury history in padel players. This relationship could identify the sport-specific deficits and inform the integration of FMS screening and injury prevention strategies within padel training. Therefore, this study aims to evaluate the correlation between FMS total score and components, and history of injury in competitive Padel players.

## 2. Materials and Methods

### 2.1. Study Design and Participants

A cross-sectional observational study was conducted with a convenience sample of competitive padel players in Jeddah. Recruitment took place between February and April 2025 through social media platforms, collaboration organizers of regional and national padel tournaments, and support from the Saudi Federation of Padel. Data collection was conducted between February and May 2025, following ethical approval. All participants were registered with the federation, trained a minimum of three sessions per week and competed regularly at regional or national levels. Player ability was classified using a UK club-based padel skill scale ranging from 1.0 to 7.0, where higher scores indicate greater playing proficiency. The scale categorizes players into beginner (1.0–2.5), intermediate (2.6–4.0), advanced (4.1–5.5), and elite (5.6–7.0) levels based on technical skills, tactical awareness, and competitive experience (see Appendix A for full description). This approach is consistent with previously published methodologies in sports performance research that emphasize standardized player classification systems for accurate comparison between groups [1,2].

Participants were classified into injured and uninjured groups based on self-reported musculoskeletal injuries sustained within the preceding 12 months. To improve reporting accuracy, a standardized injury definition was provided, whereby an injury was defined as any musculoskeletal event that (i) occurred during training or competition, (ii) required medical attention, (iii) prevented completion of the training session or match, and (iv) resulted in a minimum of four weeks of time-loss from sport.

Injury data were collected using a structured self-report questionnaire and were not independently verified through medical records. Participants were classified as ‘injured’ if they reported at least one injury meeting the defined criteria within the 12-month recall period. Multiple or recurrent injuries were not analyzed separately, and classification was based on the presence or absence of at least one qualifying injury. No distinction was made between acute and overuse injuries or between different anatomical regions for the purpose of group classification. Inclusion criteria were: (1) being male aged > 18 years, (2) training in padel >3 sessions per week, (3) No acute injury within the preceding 4 weeks, (4) providing informed consent, (5) Arabic or English language proficiency. Players with major surgery within previous 3 months or neurological, cardiovascular conditions affecting participation were excluded.

### 2.2. Instrument

An online questionnaire was used to collect demographic information, training history and self-reported injury. A calibrated anthropometric scale was used for height and weight measurement. All assessments were conducted in the Therapeutic Exercises Laboratory at the Faculty of Medical Rehabilitation Sciences, King Abdulaziz University, Jeddah, Saudi Arabia. Participants attended one in-person session, during which written informed consent was obtained prior to data collection. All procedures were performed under controlled laboratory conditions by trained assessors. Standard FMS test kit was used for the players to complete seven standard tests: Deep Squat, Hurdle Step, In-Line Lunge, Shoulder Mobility, Active Straight Leg Raise, Trunk Stability Push-up, and Rotational Stability. Each test was demonstrated prior to execution, and participants performed three recorded trials per movement. Two independent assessors scored all movements using the updated FMS scoring criteria. For bilateral tests, the lower score was recorded in accordance with FMS guidelines. Any scoring disagreements were resolved through consensus. Inter-rater reliability of FMS scoring was assessed using the intraclass correlation coefficient (ICC) based on a two-way random-effects model with absolute agreement. Reliability analyses were conducted using IBM SPSS Statistics.

All players were video recorded while performing each test, using two 12-megapixel iPad Air (10th generation) cameras for slow-motion with tripods positioned in sagittal and frontal planes (distance: 3 m; height: 70 cm). Video recordings allowed assessors to review movement patterns as needed.

### 2.3. Data Processing and Statistical Analysis

Descriptive statistics were used to summarize participant characteristics and FMS scores. Normality of continuous variables was assessed using the Shapiro–Wilk test. Given the ordinal nature of FMS scores, non-parametric statistical methods were applied. Between-group differences in FMS total and component scores were analyzed using the Mann–Whitney U test, with results reported as medians, interquartile ranges (IQR), and effect sizes (r). Given the exploratory nature of the component-level analyses, a Bonferroni correction was applied to adjust for multiple comparisons, with an adjusted significance threshold of α = 0.05/7 = 0.007. Associations between FMS total score and injury history were examined using Spearman’s rank-order correlation, reported with Spearman’s rho (ρ) and 95% confidence intervals, which were estimated using bootstrapping procedures (2000 resamples). Statistical significance was set at *p* < 0.05. Data visualization was performed using scatter plots and boxplots. All analyses were conducted using IBM SPSS Statistics v21.

A priori power analysis was conducted using G*Power (version 3.1) to evaluate the feasibility of adequately powering the planned Spearman correlation and Mann–Whitney U analyses. The analysis indicated that substantially larger samples would be required to achieve strong statistical power, particularly for between-group comparisons. Consequently, the final sample size (N = 17) reflects the maximum feasible recruitment of competitive padel players within this cohort, and findings are interpreted with greater emphasis on effect sizes and strength of associations rather than statistical significance alone.

Statistical analyses were performed independently by the authors using IBM SPSS software. The authors take full responsibility for the accuracy, integrity, and interpretation of the findings.

## 3. Results

### 3.1. Descriptive Characteristics

A total of 17 players were included in the analysis, of whom 9 were classified as injured and 8 as uninjured based on the predefined criteria. Among injured players, the most commonly reported injury locations included shoulder, elbow, knee, and ankle, reflecting the physical demands of padel.

Descriptive differences in age, BMI, and playing level are shown in Table 1. No statistically significant between-group differences were detected; however, given the small sample size, these tests should not be interpreted as evidence of equivalence between groups.

### 3.2. Normality and Score Distribution

**Figure 1:**
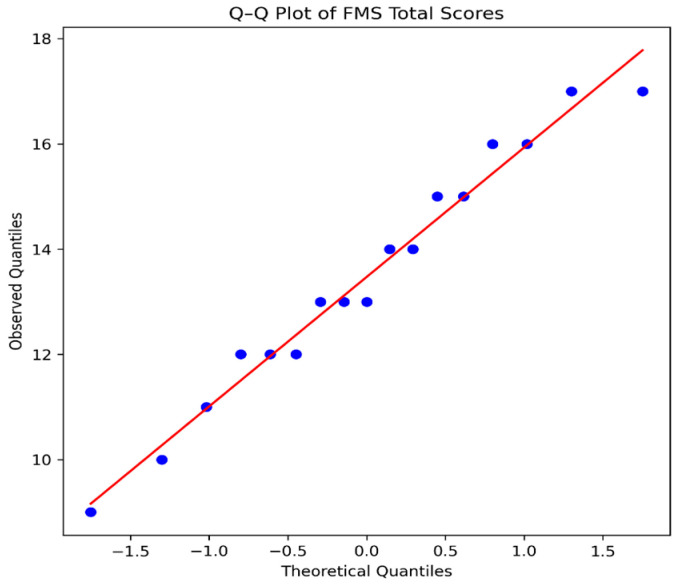
Quantile–Quantile (Q–Q) plot of Functional Movement Screen (FMS) total scores.

**Figure 2:**
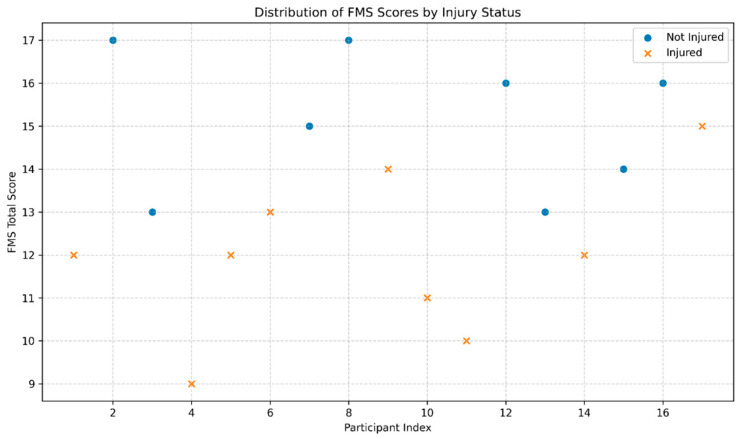
Distribution of Functional Movement Screen (FMS) total scores according to injury status.

Normality of FMS total scores was assessed using the Shapiro–Wilk test, which indicated no significant deviation from normality (W = 0.97, *p* = 0.73). Visual inspection of the histogram and Quantile–Quantile (Q–Q) plot supported this finding, with data points aligning closely with the theoretical normal distribution and no marked skewness or extreme outliers observed (Figure 1 and Figure 2).

Although the FMS total score did not significantly deviate from normality, non-parametric tests were used because FMS component scores are ordinal, injury status was dichotomous, and the sample size was small. Therefore, medians, IQRs, rank-based tests, and effect sizes were prioritized. Visual inspection of the scatterplot further demonstrated between-group clustering of FMS scores by injury status, supporting the use of distribution-free statistical methods.

### 3.3. Inter-Rater Reliability

Inter-rater reliability analysis demonstrated excellent agreement between assessors for the total FMS score (ICC = 0.97, 95% CI: 0.92–0.99). Individual component scores also showed good to excellent reliability, including Deep Squat (ICC = 0.84), In-Line Lunge (ICC = 0.94), and Shoulder Mobility (ICC = 0.98). Several components, including Hurdle Step, Active Straight Leg Raise, and Trunk Stability Push-Up, demonstrated perfect agreement (ICC = 1.00). Rotary Stability could not be analyzed due to zero variance in scores across participants (Table 2).

### 3.4. Association Between FMS Total Score and Injury History

The relationship between FMS total score and injury history was examined using Spearman’s rank-order correlation (Table 3). A significant negative association was identified (ρ = −0.703, 95% CI: −0.89 to −0.38, *p* = 0.002), indicating that players with a history of injury tended to demonstrate lower overall movement quality compared with uninjured players.

### 3.5. Comparison of FMS Scores Between Injured and Uninjured Players

The comparison of FMS total score was considered the primary analysis, whereas the seven component-level comparisons are considered exploratory secondary analyses. To assess the differences in FMS score between the two groups, Mann–Whitney U test revealed that uninjured players tended to demonstrate higher total FMS scores than injured players (U = 7.00, *p* = 0.005), with a large effect size (r = 0.68) (Table 4). This finding is consistent with the correlation analysis and suggests a potential difference in global movement quality between groups. Overall, uninjured players demonstrated higher median scores and mean ranks across most FMS components, suggesting better movement performance.

The In-Line Lunge component remained statistically significant after Bonferroni correction, with uninjured players achieving higher scores than injured players (U = 8.00, *p* = 0.004), accompanied by a large effect size (r = 0.70). Although no other individual components reached statistical significance, several tests demonstrated small-to-moderate effect sizes, including the Active Straight-Leg Raise (U = 21.50, *p* = 0.099, r = 0.40) and Trunk Stability Push-Up (U = 19.50, *p* = 0.072, r = 0.44), indicating meaningful trends toward poorer movement performance among injured players that may not have reached statistical significance due to limited statistical power. The Deep Squat, Hurdle Step, and the Shoulder Mobility tests showed small, non-significant differences between groups (*p* > 0.05). Rotary Stability demonstrated identical median scores and mean ranks in both groups (U = 36.00, *p* = 1.00, r = 0.00), indicating no association with injury history in the present sample.

## 4. Discussion

This study presents the relationship between FMS scores and history of injury among male padel players. The results suggest that lower FMS scores may be associated with injury history. Previously injured players demonstrated lower FMS scores than non-injured players, with a notable overall negative correlation (*p* = 0.002). Similarly, the most pronounced difference was observed in the In-Line Lunge task (*p* = 0.004). However, given the small sample size (N = 17), these findings should be interpreted with caution and considered exploratory. Additionally, due to the cross-sectional design, causality cannot be inferred, and it is not possible to determine whether lower FMS scores preceded injury or reflect residual impairments following injury.

These findings align with a study that reported significant lower FMS scores in previously injured young football players, particularly in the In-Line Lunge (*p* = 0.001) [11]. As the history of injury was assessed retrospectively in the current study, lower FMS scores in previously injured players may reflect residual impairments after injury rather than pre-existing injury susceptibility. Possible explanations include persistent mobility deficits, neuromuscular inhibition, compensatory movement strategies and reduced psychological readiness [12,13,14,15]. The In-Line Lunge component showed a significant difference (*p* = 0.004), with injured players demonstrating lower scores compared to uninjured counterparts. The In-Line Lunge requires multi-planer control, hip mobility, trunk stability and lumbopelvic coordination under narrow base of support [4]. Reduced scores may indicate residual limitations in lower limb mobility, balance or movement coordination. In athletes with previous ankle, knee, or hip injuries, compensatory strategies such as reduced knee flexion, trunk lean, or asymmetrical loading may negatively influence task performance. Since the injury site was not deeply investigated, it remains unclear whether reduced In-Line Lunge scores were primarily associated with lower limb injuries or post-injury movement limitations. Thus, further statistical investigation in larger, adequately powered samples is needed to clarify these relationships.

While the present study found a difference in the In-Line Lunge performance between injured and uninjured players, another study reported that asymmetry in Shoulder Mobility test was significantly associated with increased risk of injury in 12 out of 30 professional handball players using a cutoff FMS score of 13 [16]. The study suggest that the unilateral overhead demands of handball may influence FMS scores, but it questioned the validity of FMS in this sport and highlight the importance of considering injury history [16]. The present findings, on the other hand, may suggest that the FMS has potential utility for identifying movement limitations following injury, particularly in the In-Line Lunge task. Discrepancies with previous studies may be influenced by sport-specific demands, accordingly, screening should be interpreted within the sport context, while larger prospective studies are required to clarify the potential value of implementing corrective strategies in each sport.

Other studies found no significant relationship between injury and total FMS score in young tennis players and high school athletes respectively [7,8]. These varied findings suggest that the validity of FMS may vary by sport and age group. While tennis and padel are similar in movement, padel involves frequent lateral movements, which may explain the sensitivity of the In-Line Lunge in detecting injury-related deficits. Also, the young athletic population may explain the differing results, as older athletes in this study may could have greater exposure to training and competition, and potentially higher history of injury, which may influence movement quality [8]. Therefore, FMS scores may be better considered as a component of assessment rather than a stand-alone diagnostic tool; considering factors such as age and the specific demands of sport.

The present findings are indirectly supported by previous prospective studies that have evaluated the predictive value of the FMS, reporting that athletes with lower FMS scores (e.g., <14) may have a higher likelihood of sustaining injury during a competitive season [6,17]. However, these studies did not account for the influence of previous injury, which is a well-established risk factor for future injury. In contrast, the retrospective approach used in the present study examines the association between current movement quality and past injury and therefore does not allow for causal or predictive inference.

Furthermore, inconsistent findings across the literature may reflect inherent limitations of the FMS as a predictive tool. While the FMS provides a global assessment of movement quality, it may not capture sport-specific biomechanical demands or account for multifactorial contributors to injury such as training load, previous injury severity, or psychological factors. Additionally, the use of composite scoring may obscure important movement-specific deficits, and cutoff-based interpretations (e.g., score < 14) have been questioned. These limitations may partly explain the variability in its reported predictive value across different sports and populations.

While these findings provide exploratory insight into the relationship between movement quality and history of injury among competitive padel players, the small sample size (N = 17) limits the statistical power of the study and restricts the generalizability of the findings. As a result, the observed associations should be interpreted with caution, and the results should be considered exploratory rather than conclusive. Moreover, the use of self-reported injury history introduces the potential for recall bias and misclassification, as injury data were not verified through medical records. Although a clear and relatively strict injury definition and a 12-month recall period were used to improve reporting accuracy, some inaccuracies may persist. In addition, classifying participants based on the presence of at least one injury within a 12-month period may introduce heterogeneity in injury type, severity, and anatomical location. The study did not differentiate between acute and overuse injuries, nor did it account for multiple or recurrent injuries, which may influence movement patterns differently. These factors should be considered when interpreting the findings. Furthermore, although a Bonferroni correction was applied to control for multiple comparisons, this conservative approach may increase the risk of Type II error, particularly given the small sample size. Future studies involving larger, more diverse samples across different competitive levels, genders, and regions are needed to confirm these findings and strengthen the practical application of FMS as a screening tool in padel.

## 5. Conclusions

This exploratory cross-sectional study found that lower FMS total scores were associated with self-reported previous injury history among competitive male padel players. The In-Line Lunge was the only individual FMS component that differed significantly between previously injured and uninjured players after correction for multiple comparisons. These findings may reflect residual movement limitations following previous injury rather than pre-existing injury susceptibility. Given the small sample size, retrospective injury classification, and cross-sectional design, the results should be interpreted cautiously. Larger prospective studies are needed to clarify the relationship between FMS performance, injury history, and movement quality in padel athletes.

## Figures and Tables

**Table 1:** Baseline Characteristics of Uninjured and Injured Groups.

Variable	Uninjured		Injured		*p*-Value (U Mann–Whitney Test)
	M	SD	M	SD	
Age	24.83	3.25	28.31	7.08	0.31
BMI	23.58	2.38	24.91	3.69	0.48
Playing Level	5.25	0.42	5.0	0.46	0.21

BMI = Body Mass Index, M = Mean, SD = Standard Deviation.

**Table 2:** Inter-rater reliability of the FMS total and component scores.

FMS Test	ICC	95% Confidence Interval
Deep Squat	0.84	0.58–0.93
Hurdle Step	1.00	1.00
Inline Lunge	0.94	0.85–0.98
Shoulder Mobility	0.98	0.95–0.99
Active Straight Leg Raise	1.00	1.00
Trunk Stability	1.00	1.00
Rotary Stability	Zero Variance	-
FMS Total Score	0.97	0.92–0.99

FMS: Functional Movement Screen, ICC: Intraclass Correlation Coefficients.

**Table 3:** Spearman Correlation Between FMS Total Score and History of Injury.

Variables	rho	95% CI	*p*-Value	Interpretation
FMS Total ↔ Injury History	−0.703	−0.89 to −0.38	0.002	Strong negative association, statistically significant

rho: Spearman rank-order correlation coefficient, CI: Confidence Interval.

**Table 4:** Comparison of FMS Scores Between Uninjured and Injured Players.

FMS Test	Uninjured		Injured		U	*p*-Value	Effect Size (r)
	Median (IQR)	Mean Rank	Median (IQR)	Mean Rank			
Deep Squat	2.0 (1.0)	9.75	2.0 (1.0)	8.33	30.00	0.49	0.17
Hurdle Step	2.0 (1.0)	10.38	1.0 (1.0)	7.78	25.00	0.21	0.30
In-Line Lunge	2.5 (1.0)	12.5	2.0 (1.0)	5.89	8.00	0.004	0.70
Shoulder Mobility	3.0 (1.0)	10.13	2.0 (1.0)	8	27.00	0.35	0.23
Active Straight-Leg-Raise	2.0 (0.5)	10.81	2.0 (1.0)	7.39	21.50	0.099	0.40
Trunk Stability	2.0 (1.0)	11.06	2.0 (1.0)	7.17	19.50	0.072	0.44
Rotary Stability	2.0 (0.0)	9.00	2.0 (0.0)	9.00	36.00	1.00	0.00
FMS Total Score	15.5 (3.5)	12.63	12(3)	5.78	7.00	0.005	0.68

FMS: Functional Movement Screen, IQR: Interquartile Range, *p*: *p*-value, U: Mann–Whitney U statistic.

## Data Availability

The data supporting the findings of this study is available from the corresponding author upon reasonable request. Due to privacy and ethical considerations, the data is not publicly available.

## Abbreviations

The following abbreviations are used in this manuscript:

FMS	Functional Movement Screen
BMI	Body Mass Index

## Appendix A

The classification system was adapted from commonly used club-based padel frameworks and informed by established rating systems in racket sports, such as the International Tennis Number (ITN), due to the limited availability of standardized padel-specific classification systems in the literature. (For more information: https://www.itftennis.com, accessed on 3 February 2025.)

Participants were classified using a structured padel skill rating system adapted from established classification frameworks in racket sports. This approach has been widely used to ensure consistency in evaluating player ability based on technical skills, tactical awareness, and match performance.

The classification system categorizes players into four levels: Beginner, Intermediate, Advanced, and Elite. Each level reflects progressive development in stroke execution, game strategy, and competitive experience. Detailed criteria and score ranges are provided in Appendix A to enhance transparency and reproducibility of the classification process.

**Table A1:** The complete ranking system.

Category	Level	Description
Beginner (1.0–2.5)	1.0–1.5 (Exploratory)	New players: learning rules, positioning, and basic strokes
	1.6–2.5 (Foundational)	Developing fundamental skills with limited rally consistency
Intermediate (2.6–4.0)	2.6–3.5 (Developmental)	Improved control and ability to sustain rallies; emerging tactical awareness
	3.6–4.0 (Proficient)	Consistent gameplay with basic strategic understanding
Advanced (4.1–5.5)	4.1–4.8 (Competitive)	Regular competitive participation with solid technical and tactical skills
	4.9–5.5 (Expert)	Advanced techniques, effective match control, and high-level performance
Elite (5.6–7.0)	5.6–6.5 (Master)	National or semi-professional level players
	6.6–7.0 (Professional)	Professional players competing internationally
